# Factors Associated with the Early Ambulation After Elective Lumbar Spinal Surgery: A Retrospective Cohort Study

**DOI:** 10.3390/jcm15135307

**Published:** 2026-07-07

**Authors:** Busakorn Ruksouy, Tanyong Pipanmekaporn, Pathomporn Pin-on, Pichitchai Atthakomol, Piyada Boonsong

**Affiliations:** 1 Department of Anesthesiology, Phichit Provincial Hospital, Phichit 66000, Thailand; rugsouy1540@gmail.com; 2 Department of Anesthesiology, Faculty of Medicine, Chiang Mai University, Chiang Mai 50200, Thailand; pinon.pathomporn@gmail.com (P.P.-o.); piyadaster@gmail.com (P.B.); 3 Department of Biomedical Informatics and Clinical Epidemiology, Faculty of Medicine, Chiang Mai University, Chiang Mai 50200, Thailand; p.atthakomol@gmail.com; 4 Department of Orthopaedics, Faculty of Medicine, Chiang Mai University, Chiang Mai 50200, Thailand

**Keywords:** early ambulation, lumbar spinal fusion, associated factors, clinical outcomes

## Abstract

**Background**: Early ambulation (EA), mobilizing within 24 h, is a cornerstone of recovery after lumbar spine surgery. Clinical evidence suggests that EA is strongly associated with superior postoperative outcomes, including accelerated functional recovery, reduced complications, and a significantly shorter length of hospital stay. Despite these benefits, there remains a critical gap in the literature regarding the specific patient and surgical factors that influence a patient’s ability to achieve early mobilization. **Objective**: To identify factors associated with EA and assess its relationship with postoperative recovery after elective lumbar spinal fusion. **Methods**: A retrospective cohort study was conducted at Phichit Provincial Hospital between 2017 and 2024. Demographic, anesthesia, and surgical data were collected. Univariable and multivariable logistic regression analyses were performed to identify independent factors associated with EA. **Results**: Of 581 patients, 332 were in the EA group (57.2%), and 249 (42.8%) were in the delayed ambulation (DA) group. Independent factors associated with EA included American Society of Anesthesiologists (ASA) class I–II (OR 1.60, 95% CI 1.05–2.43, *p* = 0.027), anesthetic time < 200 min (OR 2.04, 95% CI 1.25–3.35, *p* = 0.005), and intravenous parecoxib administration (OR 3.45, 95% CI 2.34–5.18, *p* < 0.001). The EA group demonstrated significantly lower complication rates (3.9% vs. 20.4%, *p* < 0.001), shorter hospital stays (7 [IQR 6–9] vs. 9 [IQR 8–12] days, *p* < 0.001), and reduced total hospitalization costs ($1958 [IQR $933–$2966] vs. $2815 [IQR $1019–$3555], <0.001). No significant difference in 30-day readmission rates was observed. **Conclusions**: EA after elective lumbar spinal fusion was associated with better postoperative recovery. Factors independently associated with EA included lower ASA class, shorter anesthetic duration, and postoperative intravenous parecoxib use.

## 1. Introduction

Lumbar spine surgery is among the most painful surgical procedures and can increase the risk of chronic postoperative pain and subsequent opioid dependence. Common lumbar interventions—including laminectomy, discectomy, and spinal fusion—are performed to alleviate back pain, radiculopathy, and neurological deficits resulting from degenerative disc disease, spinal stenosis, or spondylolisthesis [1,2,3,4,5,6]. Importantly, the definition of a successful elective outcome has expanded beyond intraoperative technical performance and postoperative radiological confirmation. Contemporary measures of success emphasize the pace of functional recovery and improvements in patient-reported outcomes, in addition to technical and imaging-based metrics [7]. However, variability in surgical techniques and perioperative care pathways across institutions contributes to differences in postoperative outcomes, including length of hospital stay and the trajectory of functional recovery [8,9,10,11,12,13,14,15].

Early ambulation (EA) is a key component of Enhanced Recovery After Surgery (ERAS) pathways in lumbar spine surgery and generally refers to mobilization within 24 h after surgery. EA helps preserve muscle strength, improve respiratory function, and support psychological well-being [16,17,18,19]. In contrast, prolonged bed rest is strongly associated with severe postoperative complications, including deep vein thrombosis and postoperative ileus [18,19,20,21,22,23]. Although the benefits of early mobilization are widely recognized, consistent implementation remains challenging. Mobilization is often delayed by a complex interplay of patient-, procedure-, and system-level factors. Key determinants heavily investigated include patient demographics and baseline health status (age, gender, and ASA physical status), surgical variables (operative time, estimated blood loss, number of surgical levels, and instrumentation), as well as analgesic techniques managed by the care team [21,22,23,24].

Understanding the exact predictors of delayed vs. early ambulation allows perioperative teams to risk-stratify patients pre-operatively and design targeted interventions (such as optimized multimodal analgesia or dedicated physical therapy pathway. Therefore, the objective of this study was to identify factors associated with EA and evaluate postoperative outcomes following elective lumbar spinal fusion.

## 2. Materials and Methods

### 2.1. Study Design and Patient Cohort

The study protocol was approved by the Institutional Review Board (IRB) of Phichit Provincial Hospital (protocol number 0194/2022). The requirement for informed consent was waived due to the retrospective nature of the study. Data extraction and case reviews were performed by three nurse anesthetists, each with at least five years of experience in perioperative and postoperative monitoring. Before commencement, the principal anesthesiologist (BR) standardized all variables, including baseline data, anesthetic and surgical details based on the International Classification of Diseases, 10th Revision (ICD-10), and pain management protocols. The study period, EA definition, and clinical outcome metrics were strictly defined to maintain consistency across all reviews. We retrospectively reviewed medical records of adults ≥ 18 years undergoing elective lumbar spine surgery at Phichit Provincial Hospital, a 400-bed tertiary care facility in Northern Thailand, between 2017 and 2024. Surgical indications included lumbar spinal stenosis, lumbar disc herniation, and degenerative spondylolisthesis. All procedures consisted of open posterior decompression with posterolateral fusion, with or without instrumentation. Patients were excluded if they had a history of spine trauma, malignancy of spine, preoperative neurological deficits, or underwent Minimally Invasive Surgery (MIS) using endoscope- or microscope-assisted techniques for the current hospitalization. Additional exclusion criteria included the requirement for postoperative mechanical ventilation, a history of Failed Back Surgery Syndrome (FBSS)—persistent or recurrent low back or leg pain after spinal operations [25], death within 24 h after surgery, or postoperative transfer to another hospital. Revision procedures were excluded because prior spinal surgery may substantially affect operative complexity, postoperative recovery, pain levels, and ambulation patterns. MIS procedures were excluded to maintain a more homogeneous study population, as these techniques are associated with reduced tissue trauma, lower postoperative pain, and faster postoperative recovery compared with conventional open lumbar fusion.

### 2.2. Anesthesia, Surgery, and Perioperative Care

On the evening before surgery, patients underwent routine preoperative assessment by trained nurse anesthetists. Relevant clinical data were then relayed to the attending anesthesiologists to ensure tailored and appropriate care. In accordance with institutional protocols, antiplatelet medications were withheld before surgery; specifically, aspirin and clopidogrel were discontinued at least 7–10 and 5–7 days preoperatively, respectively, unless clinically contraindicated. Warfarin was typically discontinued five days before surgery with normalization of the International Normalized Ratio (INR) to <1.5 before the procedure, and bridging therapy was considered when indicated based on individual thromboembolic risk, while low-molecular-weight heparin was discontinued at least 24 h before surgery [26]. All procedures were performed under general anesthesia according to institutional practice. Intraoperative variables collected for analysis included anesthetic duration, estimated blood loss, and perioperative analgesic administration. Estimated blood loss was determined using suction canister volumes (excluding irrigation), surgical sponge counts, and visual assessment. After the skin incision, patients received tranexamic acid at a dose of 10–15 mg/kg as an intravenous drip, followed by a continuous infusion at 50 mg/h, as requested by the attending surgeon when no contraindications were present.

All patients underwent elective open lumbar spinal fusion through a conventional posterior midline approach for degenerative lumbar disorders. Under general anesthesia, patients were positioned prone, and the operative level was confirmed fluoroscopically. A posterior midline incision was made, followed by subperiosteal dissection to expose the posterior spinal elements. Neural decompression was performed as indicated, including laminectomy, facetectomy, and/or foraminotomy to adequately decompress the neural structures. Posterolateral fusion was achieved by decorticating the transverse processes and adjacent posterior elements. Autologous local bone graft obtained during decompression was placed over the prepared fusion bed. Pedicle screw instrumentation (Hermith GmbH, Munich, Germany) was used when indicated to provide segmental stability. 

All surgeries were performed by one of ten board-certified spine surgeons with more than five years of specialized experience in spinal surgery. Each individual procedure was conducted by a single attending surgeon, ensuring consistency in surgical expertise across cases. The decision to use instrumentation was made at the discretion of the operating surgeon based on intraoperative findings and patient-specific considerations.

Upon completion of surgery, all patients were extubated and transferred to the post-anesthesia care unit (PACU) for routine monitoring. Postoperative pain was assessed using the Numerical Rating Scale (NRS; 0–10). Patients were discharged to the ward once they achieved adequate recovery, defined by a Modified Aldrete Score > 9 and mild pain (NRS 0–3). Postoperative analgesia consisted of intravenous opioids as needed and intravenous parecoxib (Dynastat, Pfizer, Kent, UK) when no contraindications were present, including active bleeding or excessive postoperative drain output. The first dose of parecoxib was administered upon arrival at the ward and continued every 12 h for 48 h. Patients reporting moderate-to-severe pain (NRS ≥ 4) received intravenous morphine (0.1 mg/kg) every 4–6 h as required. Pain scores were recorded every 6 h in the hospital ward. Total opioid consumption during the first 96 postoperative hours was converted to morphine milligram equivalents (MME). The drainage tube was removed approximately on postoperative day 3, when the blood drainage volume was either 30 mL within eight hours.

### 2.3. Hospital Ambulation Protocol

Patients were assessed by staff nurses trained in postoperative spine care and mobilization protocols, or by physiotherapists, prior to initiating mobilization. The assessment was performed approximately 6–8 h postoperatively. Mobilization was initiated only when patients met all of the following criteria: (1) full consciousness, defined as a Glasgow Coma Scale (GCS) score ≥ 14; (2) hemodynamic stability, defined as absence of orthostatic hypotension and stable vital signs (systolic blood pressure > 90 mmHg, heart rate < 100 bpm, and oxygen saturation > 94% on room air); and (3) acceptable pain control, defined as a numeric rating scale (NRS) pain score at rest < 4 (4) Surgical drains were secured with no evidence of bleeding.

Mobilization followed a structured, stepwise protocol that began with log-rolling in bed and progressed to sitting at the bedside while maintaining proper spinal alignment with lumbosacral support, then standing, and finally ambulating out of bed with or without assistance as tolerated [27,28]. The EA was defined as the ability to get out of bed and walk within 24 h after surgery; ambulation occurring after 24 h was classified as delayed ambulation (DA).

### 2.4. Data Source and Measurement

Three trained nurse anesthetists independently reviewed demographic data, anesthetic records, and surgical details. Demographic and clinical data included age, gender, BMI, smoking status, and underlying medical conditions such as Chronic Obstructive Pulmonary Disease (COPD), Coronary Artery Disease (CAD), diabetes, and Chronic Kidney Disease (CKD). Additional preoperative variables included the ASA classification and INR. Surgical variables recorded were the type of spinal fusion and number of fusion levels, duration of surgical procedures (defined as the duration from skin incision to skin closure), duration of anesthesia (defined as the duration from induction of anesthesia to extubation, including anesthetic management until transfer from the operating room), estimated blood loss, administration of tranexamic acid, and intraoperative opioid consumption.

Postoperative outcomes included opioids used at the PACU, opioids used within the first 96 postoperative hours, and NRS scores were assessed every six hours for a total duration of 96 h postoperatively. Additional outcomes included the amount of surgical drain output, assessed until clinical indication for drain removal, and the incidence of severe Postoperative Nausea and Vomiting (PONV), monitored over a 96 h postoperative period. Postoperative data included the Length of Stay (LOS), total hospital cost, and 30-day readmission rate. All treatment-related expenses—including medications, surgical supplies, anesthesia services, laboratory and radiologic investigations, nursing care, rehabilitation, and room charges—were incorporated into the calculation of total hospital cost. Total costs were compared between groups, with higher costs influenced not only by direct treatment expenses, but also by increased healthcare resource utilization associated with prolonged LOS and postoperative complications. Postoperative complications recorded urinary retention, constipation, hypovolemic shock, acute myocardial ischemia, pulmonary embolism (PE), atelectasis, pneumonia, hematoma, surgical site infection, implant displacement (defined as postoperative migration, loosening, or malposition of spinal implants identified on imaging), and the need for reoperation (defined as any unplanned return to the operating room related to the initial lumbar spine surgery).

### 2.5. Clinical Outcomes

The primary outcome of this study was to identify factors associated with EA within 24 h following lumbar spinal fusion surgery. The secondary outcomes involved were comparing the incidence of postoperative complications, LOS, 30-day readmission rate, and overall hospitalization costs between EA and DA groups.

### 2.6. Statistical Analysis

All statistical analyses were performed using STATA version 16 (StataCorp LP, College Station, TX, USA). A two-sided *p*-value of less than 0.05 was considered statistically significant. Categorical variables were presented as frequencies and percentages. These variables were analyzed using Fisher’s exact test. Continuous variables were expressed as means with Standard Deviations (SD). The normality of the data distribution was assessed using the Shapiro–Wilk test. Student’s *t*-test was used for normally distributed data. Non-normally distributed data were presented as medians with interquartile ranges (IQR) and compared using the Wilcoxon rank-sum test. A *p*-value of <0.05 was considered statistically significant.

Preliminary data from Phichit Provincial Hospital between January and June 2021 indicated that EA was achieved in approximately 60% of patients, while DA occurred in 40%, corresponding to an approximate ratio of 3:2. The sample size was calculated based on a review of medical records assessing factors influencing EA, using STATA software with a significance level (α) of 0.05 (two-sided test), a power of 80%. A total sample size of 225 patients was estimated to be required, comprising 135 patients in the EA group and 90 patients in the DA group.

To identify independent risk factors associated with EA, both univariate and multivariable logistic regression analyses were conducted. Variables with a *p*-value < 0.1 in univariable logistic regression analysis were considered for inclusion in the multivariable model. The results were presented as ORs with 95% confidence intervals (CI), and statistical significance was set at *p* < 0.05. The analysis and reporting followed the Strengthening the Reporting of Observational Studies in Epidemiology (STROBE) checklist guidelines [29]. To assess the robustness of the primary findings and minimize potential residual confounding, a sensitivity analysis was performed in a more homogeneous subgroup of patients undergoing ≥3-level instrumented lumbar fusion. Postoperative outcomes and factors associated with early ambulation were re-evaluated using univariable and multivariable logistic regression analyses.

## 3. Result

**Figure 1:**
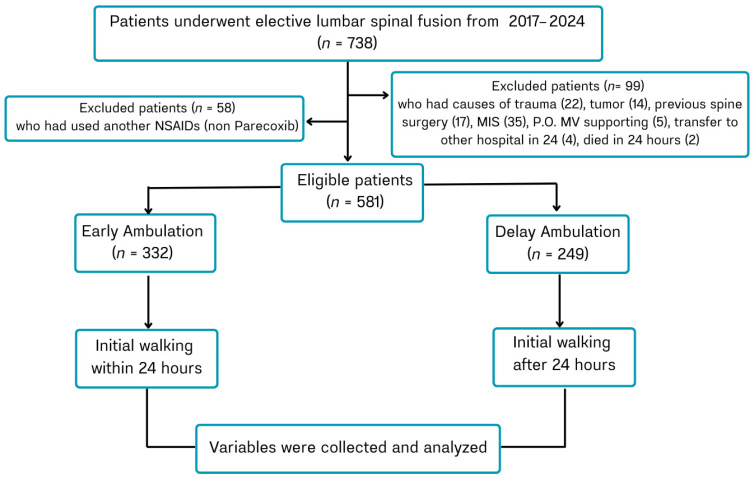
Study Flow Chart.

Of the initial 738 patients assessed for eligibility over eight years between January 2017 and December 2024, 157 were excluded based on the study’s exclusion criteria. A total of 581 patients were included in the final analysis, with 332 classified in the EA group and 249 in the DA group (Figure 1). The incidence of EA and DA was 57.1% and 42.9%, respectively.

Table 1 presents the demographic and baseline characteristics, as well as the anesthetic and surgical details, of patients undergoing elective lumbar spinal fusion. The EA was significantly associated with younger, ASA class I–II, non-instrumented fusion, and procedures involving fewer than three spinal levels. The median anesthetic time in the EA group was 200 min [IQR, 160–315], compared with 280 min [IQR, 180–415] in the DA group. Intraoperative blood loss was comparable between groups, with a median of 500 mL [IQR, 450–500] in the EA group and 500 mL [IQR, 500–1300] in the DA group. Patients in the EA group required significantly lower median cumulative opioid doses within the first 96 postoperative hours compared to the DA group, 8 MME [IQR, 5–13] vs. 13 MME [IQR 10–16], *p* < 0.001.

All patients underwent open lumbar decompression and posterolateral fusion for degenerative lumbar disorders, with pedicle screw instrumentation in 60% of cases and without instrumentation in 40%. The median number of operated spinal levels was 2 [IQR 2–3].

Table 2 and Table 3 summarize the univariable and multivariable analyses of factors associated with early ambulation following elective lumbar spinal fusion. Table 3 showed that ASA class I–II (OR 1.60, 95% CI 1.05–2.43, *p* = 0.027), anesthetic time < 200 min (OR 2.04, 95% CI 1.25–3.35, *p* = 0.005), and IV parecoxib administration (OR 3.45, 95% CI 2.34–5.08, *p* < 0.001) were significant predictors of EA after lumbar spine surgery.

The incidence of postoperative complications within 96 h was significantly lower in the EA group (13 of 332, 3.9%) compared to the DA group (51 of 249, 20.4%) (*p* < 0.001). The DA group demonstrated significantly higher rates of urinary tract infection (5.2% vs. 0.9%, *p* = 0.003), pulmonary atelectasis (1.6% vs. 0%, *p* = 0.034), pneumonia (4.0% vs. 0%, *p* < 0.001), surgical site infection (4.8% vs. 0%, *p* < 0.001), displacement of implant (2.0% vs. 0%, *p* < 0.001), and reoperation (4.4% vs. 0%, *p* < 0.001).

In the DA group, three cases of postoperative hematoma and five cases of implant displacement occurred on the first postoperative day, before initiation of ambulation. These cases presented with lower limb numbness without motor deficit. Importantly, no incidences of hematoma or implant-related symptoms were reported in the EA group. The EA group had a significantly shorter LOS (7 vs. 9 days; *p* < 0.001) and lower hospitalization costs ($1958 vs. $2815; *p* < 0.001), without an increase in the 30-day readmission rate (1.5% vs. 2.0%; *p* = 0.751) (Table 4).

Sensitivity analyses restricted to patients undergoing ≥ 3-level instrumented lumbar fusion demonstrated findings consistent with the primary analysis (Supplementary Tables S2–S4). In the multivariable analysis, ASA class I–II (OR 2.27, 95% CI 1.02–5.03, *p* = 0.045), anesthetic time < 300 min (OR 2.59, 95% CI 1.32–5.06, *p* = 0.005), and postoperative intravenous parecoxib administration (OR 12.36, 95% CI 5.88–26.06, *p* < 0.001) remained independently associated with early ambulation (Table S4). Patients undergoing ≥ 3-level instrumented lumbar fusion in the EA group had lower opioid consumption, fewer overall complications, a shorter length of stay, and lower hospitalization costs than those in the DA group (Table S2).

## 4. Discussion

This study identified three related factors associated with EA within 24 h following elective lumbar spinal fusion. ASA class I–II, anesthetic time < 200 min, and postoperative intravenous parecoxib use were independently associated with EA. Other variables—such as age, sex, non-instrumented fusion, number of fused segments, and blood loss < 500 mL—showed favorable trends but were not statistically significant after multivariable logistic regression analysis. Our findings demonstrate that EA is a critical determinant of superior postoperative recovery, significantly enhancing clinical outcomes for patients undergoing elective lumbar spinal fusion. Patients in the EA group also demonstrated more favorable postoperative recovery, including a shorter LOS and fewer complications than those in the DA group; this difference was driven primarily by a lower frequency of respiratory, urinary, and surgical site infections. EA is a core element of ERAS protocols across multiple orthopedic procedures. Following lumbar fusion, EA may directly support functional recovery after neural decompression, reduce paraspinal muscle spasm, and alleviate radicular symptoms, representing a spine surgery; this specific mechanism was not observed in extremity orthopedic surgery [14,21,22,23].

Our findings regarding ASA status are supported by previous evidence. Culcasi et al. demonstrated that ASA physical status ≥ 3 was independently associated with a lower likelihood of achieving EA following spinal arthrodesis, suggesting that patients with greater comorbidity burden and reduced physiological reserve may experience slower postoperative recovery and mobilization [23]. Consistent with previous reports, our findings indicate that patients with ASA class I–II were significantly more likely to achieve EA within 24 h. Furthermore, Bronheim et al. reported that ASA class ≥ 3 was associated with a decreased likelihood of EA, prolonged LOS, and increased healthcare resource utilization [30]. Taken together, these findings highlight ASA status a key indicator of physiological reserve influencing both recovery trajectory and EA. In our study, patients classified as ASA class III frequently had multiple comorbidities, including hypertension, dyslipidemia, diabetes mellitus, and diabetes mellitus, representing a high burden of non-communicable diseases that may adversely affect postoperative recovery and limit EA.

In our study, anesthetic time was chosen in place of operative time as a surrogate of operative factors, as it more comprehensively captures the perioperative course, particularly the emergence from anesthesia. The association between shorter anesthetic duration and EA observed in our study is supported by prior evidence, as Culcasi et al. reported that longer operative duration (mean approximately 230 min) was independently associated with slower postoperative mobilization following spinal arthrodesis (*p* < 0.001), suggesting that increased surgical burden may hinder postoperative mobilization [23]. Although their analysis focused on operative time, this parameter is closely related to anesthetic duration in clinical practice. Similarly, Lovecchio et al. demonstrated that shorter operative duration was associated with reduced LOS, reflecting improved postoperative recovery and a greater likelihood of achieving EA after surgery [31]. Consistent with these findings, our results showed that shorter anesthetic time (<200 min) was independently predictor of a shorter time to ambulation, specifically within the first 24 h post-surgery.

Effective postoperative pain management plays a crucial role in facilitating EA and functional recovery after spine surgery by reducing pain-related movement limitation and opioid-related adverse effects. In the study by Chiu et al., ambulation was assessed indirectly through functional recovery, as reflected by improvements in activities of daily living and mobility measured using the Barthel Index (BI). Although ambulation was not directly defined as a time-based outcome, intravenous parecoxib (40 mg every 12 h for 48 h) significantly improved early postoperative recovery following lumbar spine surgery. Specifically, compared with morphine-based patient-controlled analgesia, parecoxib was associated with lower postoperative pain scores and reduced opioid consumption [24]. Furthermore, patients receiving parecoxib demonstrated earlier recovery of mobility and activities of daily living, with greater BI score improvements at 48 h (increase of 4.36 points, *p* = 0.04) and 72 h (increase of 6.26 points, *p* < 0.01). These findings suggest that improved postoperative analgesia may facilitate EA. Importantly, parecoxib has a favorable safety profile, with no significant increase in adverse events such as gastrointestinal complications, renal dysfunction, or bleeding compared with opioid-based regimens [24]. Consistent with these findings, our previous study demonstrated that postoperative intravenous parecoxib, administered using a comparable regimen, was independently associated with EA following lumbar spinal fusion [24]. Our previous study showed that postoperative intravenous parecoxib significantly promoted early ambulation after elective lumbar spine surgery by shortening the time to mobilization (22.7 vs. 33.1 h, *p* < 0.001). Patients who received parecoxib also had lower opioid consumption during the first 96 postoperative hours (9.6 vs. 13.2 MME, *p* < 0.001) and a shorter hospital stay (6.1 vs. 9.6 days, *p* < 0.001) compared with those who did not receive parecoxib. This may because parecoxib supports postoperative mobilization by improving pain control, enhancing functional recovery, and reducing opioid-related adverse effects such as nausea, sedation, and dizziness [32]. Taken together, the results of our study support the role of parecoxib as an important component of multimodal analgesia strategies aimed at promoting EA after lumbar spine surgery, provided that no contraindications or clinically significant postoperative bleeding are present.

The association between EA and reduced postoperative complications observed in our study is supported by previous evidence. Zakaria et al., in a large multicenter cohort from the MSSIC, demonstrated that ambulation on the day of surgery—defined as getting out of bed and walking on the day of surgery after recovery from anesthesia—was associated with a significant reduction in overall morbidity and adverse events without increasing the risk of major complications [22]. Similarly, Wang et al. reported that, compared with DA, EA within 24 h in elderly patients undergoing lumbar fusion was associated with significantly lower rates of pulmonary complications (3.5% vs. 10.8%, *p* = 0.028) and urinary tract infections (2.7% vs. 8.1%, *p* = 0.041) [21]. Likewise, Owaicho et al. reported that, compared with patients who achieved EA on the day of surgery, those with DA had higher rates of perioperative complications (54.8% vs. 23.8%, *p* = 0.01) and a longer LOS (8.1 vs. 5.3 days, *p* = 0.01). The DA was also associated with poorer functional independence at discharge (*p* = 0.03) [17]. Taken together, these findings reinforce the role of EA as an effective strategy to reduce postoperative morbidity and enhance recovery following lumbar spine surgery. 

In our study, EA was significantly associated with a shorter LOS, with the EA group demonstrating a median LOS of seven days versus nine days in the DA group (*p* < 0.001). These findings are consistent with previous studies. Owoicho et al. reported that EA reduced LOS in elderly patients undergoing thoracic and/or lumbar spine fusion for adult degenerative scoliosis (approximately four vs. six days) [17]. Similarly, Pitter et al., in a nationwide cohort, found that DA following primary surgery for adult spinal deformity was associated with significantly prolonged hospitalization (median LOS: 7 vs. 12 days) [33]. In the present study, the 30-day readmission rate was evaluated as an outcome because unplanned readmissions may reflect postoperative complications, delayed recovery, or unresolved functional limitations, thereby serving as a clinically relevant indicator of early postoperative outcomes and the overall quality of perioperative care. In our cohort, EA was not associated with an increased 30-day readmission rate. However, this finding should be interpreted cautiously, as the sample size may have been insufficient to detect small differences, and larger studies are needed to further clarify the relationship between EA and readmission risk. In the present study, EA was associated with a significant reduction in total hospital cost compared with the DA group, representing a cost difference of approximately $832 per patient. This finding is consistent with evidence from enhanced recovery literature. A 2025 systematic review and meta-analysis by Büchel et al., which evaluated studies published over the review search period across various spinal surgery sub-specialties and included 16 studies (n = 16), reported that 15 studies (93.8%) demonstrated reduced hospital expenditures, with a median cost reduction of approximately $1161 per case [34]. Similarly, a 2025 evidence synthesis by Sescu et al. based on multiple systematic reviews and meta-analyses of ERAS protocols in spine surgery demonstrated a mean cost reduction of approximately $1029 per patient [35].

Our findings showed that ASA class I–II, anesthetic time < 200 min, and postoperative intravenous parecoxib were key factors associated with ambulation within 24 h. An anesthetic time of 200 min was used as the cutoff to stratify EA status. These results suggest that preoperative optimization of comorbidities, correction of abnormal laboratory findings, and appropriate risk stratification may improve physiological reserve. In patients with ASA class ≥ III, minimizing anesthetic time, efficient intraoperative management, and multimodal analgesia may be particularly important. Optimizing these modifiable perioperative factors may promote earlier recovery and postoperative mobilization after lumbar spine surgery. As a regional hospital, the implementation of EA may be influenced by organizational and resource constraints. In our setting, prolonged anesthetic duration may partly reflect logistical delays, such as coordination for patient repositioning and shared operating room staff. Although EA after spine surgery may offer clinical benefits, it should be implemented with appropriate precautions based on patient condition, staffing, and available resources. Advanced support, such as Somatosensory Evoked Potentials (SSEP), Motor Evoked Potentials (MEP), and MIS techniques, is more commonly available in larger centers. Therefore, adoption should be tailored to each institution’s readiness and local care pathways.

### 4.1. Strengths

First, data were collected over eight years, allowing for a large and clinically diverse sample. Second, a key strength of this study was the concurrent evaluation of anesthetic time, whereas most previous studies have focused primarily on operative time alone. Anesthetic time captures the entire perioperative process, beginning with the induction of anesthesia and extending to extubation, encompassing critical activities such as anesthesia-related procedures, patient positioning, and preparation and readiness of the fluoroscopy equipment before surgical incision. Recognizing the importance of organizational management in regional hospitals that may influence EA. Third, the inclusion of total hospitalization costs provides important insight into the economic impact of EA. While EA was associated with reduced healthcare expenditures, the actual cost of care often exceeded reimbursement from healthcare coverage schemes, reflecting a financial burden on hospitals. These findings suggest that promoting EA may help reduce resource utilization and partially alleviate institutional financial strain. To our knowledge, there is limited published literature from developing countries evaluating hospitalization costs in the context of EA after spine surgery. Finally, the use of a 30-day follow-up period enabled a comprehensive assessment of short-term postoperative outcomes, including complications and readmission rates, thereby allowing meaningful comparison between EA and DA groups. Thirty-day readmission was determined through review of medical records to identify any hospital admissions occurring within 30 days after the initial lumbar spine surgery. This timeframe is consistent with the American College of Surgeons-National Surgical Quality Improvement Program (ACS-NSQIP) standards and prior spine literature, which consider 30-day readmission a reliable and clinically relevant indicator of postoperative recovery [36].

### 4.2. Limitations

First, an important limitation of this study was its retrospective design, which may have resulted in missing data, inconsistent recording of clinical variables, and non-uniform assessment of functional outcomes, particularly BI measurements before and after surgery. In addition, incomplete documentation of preoperative pain status—specifically whether pain was chronic or acute—may have influenced postoperative pain responses and mobility outcomes. Future prospective studies, particularly Randomized Controlled Trials (RCTs) with standardized data collection, are warranted to validate these findings and more robustly assess the impact of EA on functional recovery. Second, as a regional hospital, physiotherapy services for postoperative ambulation may not be available outside regular working hours or during weekends, potentially DA. Addressing this gap through extended or on-call physiotherapy services may improve the timeliness of ambulation, enhance patient recovery, and reduce complications, while also contributing to more efficient use of hospital resources and overall cost reduction. A third limitation was the single-center design, which may restrict the generalizability of our findings because patient populations and perioperative practices vary between hospitals. A future multicenter randomized controlled trial would help address these limitations and strengthen the evidence base for EA.

## 5. Conclusions

Successful EA (within 24 h) following elective lumbar spinal fusion was independently associated with an ASA class I–II, an anesthetic duration of less than 200 min, and the administration of intravenous parecoxib for postoperative analgesia. Patients in the EA group experienced fewer complications, decreased hospital stays, and reduced total costs, while maintaining 30-day readmission rates comparable to those with DA. By promoting EA, these findings may help improve recovery while reducing hospitalization costs and resource utilization.

## Figures and Tables

**Table 1:** Demographic Data of Patients with Early and Delayed Ambulation Groups.

Associated Factors	EA Group (*n* = 332)	DA Group (*n* = 249)	*p*-Value
Preoperative data			
Age (years)	59.55 ± 8.53	61.31 ± 8.47	0.014 †
Male	83 (33.33)	89 (26.81)	0.098
BMI (kg/m^2^)	25.39 ± 3.88	25.59 ± 4.44	0.560
Smoker	35 (10.54)	36 (14.4)	0.161
ASA class 1–2	248 (74.70)	159 (63.86)	0.006 *
Underlying diseases			
COPD	5 (1.51)	9 (3.61)	0.110
CAD	4 (1.20)	2 (0.80)	0.705
DM	80 (24.10)	52 (20.88)	0.370
CKD	18 (5.42)	12 (4.82)	0.851
Baseline INR	1.00 ± 0.07	1.01 ± 0.07	0.205
Intraoperative data			
Type of operation			0.001 *
BG fusion only	158 (47.59)	85 (34.14)	
BG and instrument fusion	174 (52.41)	164 (65.86)	
Spinal fusion			0.001 *
<3 levels	196 (59.04)	111 (44.58)	
≥3 levels	136 (40.96)	138 (55.42)	
Operative time (minutes)	180 (140–290)	260 (160–390)	<0.001 ‡
Anesthetic time (minutes)	200 (160–315)	280 (180–415)	<0.001 ‡
Blood loss (mL)	500 (450–500)	500 (500–1300)	<0.001 ‡
IV infusion of tranexamic acid	189 (56.95)	158 (63.45)	0.124
Amount of intraop. opioids consumption (MME)	12 (10–15)	12 (10–15)	0.694
Postoperative data			
Amount of opioid requirement at PACU (MME)	3 (0–5)	3 (0–5)	0.971
Received IV parecoxib administration	160 (48.19)	56 (22.49)	<0.001 *
Amount of opioid used in 96 h (MME)	8 (5–13)	13 (10–16)	<0.001 ‡
P.O. drain in 96 h (mL)	192.74 ± 79.02	195.02 ± 87.48	0.743

Note: Data are presented as frequency (percentage), mean ± SD, or median (IQR). * *p*-value from Fisher’s exact test; † *p*-value from Student’s *t*-test; ‡ *p*-value from Wilcoxon rank-sum test. *p* < 0.05 indicates statistical significance. Abbreviations: SD (Standard Deviation), BMI (body mass index), INR (International Normalized Ratio), ASA (American Society of Anesthesiologist), COPD (chronic obstructive pulmonary disease), DM (diabetic mellitus), CAD (coronary artery disease), CKD (chronic kidney disease), BG (bone graft), mL (milliliter), IV (Intravenous), intraop. (intraoperative), PACU (Post-Anesthesia Care Unit), MME (milligram of intravenous morphine equivalents), P.O. (Postoperative).

**Table 2:** Univariable Analysis of Factors Associated with Early Ambulation Following Elective Lumbar Spinal Fusion.

Associated Factors	uOR	95% CI of Odds Ratio	*p*-Value
Age ≤ 65 years	1.44	0.99–2.09	0.045 *
Male	0.73	0.50–1.07	0.099
ASA class 1–2	1.67	1.15–2.43	0.006 *
BG spinal fusion only	1.75	1.23–2.50	0.001 *
Spinal fusion < 3 levels	1.79	1.27–2.53	0.001 *
Anesthetic time < 200 min	2.16	1.51–3.09	<0.001 *
Intraoperative EBL < 500 mL	1.98	1.33–2.94	<0.001 *
Postoperative intravenous parecoxib use	3.21	2.19–4.72	<0.001 *

Note: * *p* < 0.05 indicates statistical significance. Abbreviations: uOR (univariable odds ratio), CI (Confidence Interval), ASA (American Society of Anesthesiologists), BG (bone graft), EBL (estimated blood loss), mL (milliliter).

**Table 3:** Multivariable Analysis of Factors Associated with Early Ambulation Following Elective Lumbar Spinal Fusion.

Associated Factors	mOR	95% CI of Odds Ratio	*p*-Value
Age ≤ 65 years	1.35	0.90–2.03	0.146
Male	0.76	0.51–1.14	0.189
ASA class 1–2	1.60	1.05–2.43	0.027 *
BG spinal fusion only	0.88	0.52–1.46	0.614
Spinal fusion < 3 levels	1.25	0.84–1.87	0.265
Anesthetic time < 200 min	2.04	1.25–3.35	0.005 *
Intraoperative EBL < 500 mL	1.46	0.93–2.28	0.098
Postoperative intravenous parecoxib use	3.45	2.34–5.08	<0.001 *

Note: * *p* < 0.05 indicates statistical significance. Abbreviations: mOR (multivariable odds ratio), CI (Confidence Interval), ASA (American Society of Anesthesiologists), BG (bone graft), EBL (estimated blood loss), mL (milliliter).

**Table 4:** Comparison of postoperative outcomes of patients who had early and delayed ambulation after elective lumbar spinal fusion.

Postoperative Outcomes	EA Group (*n* = 332)	DA Group (*n* = 249)	*p*-Value
Overall Complications	13 (3.9)	51 (20.4)	<0.001 *
Urinary infection	3 (0.9)	13 (5.2)	0.003 *
Constipation	9 (2.7)	11 (4.4)	0.167
Hypovolemic shock	3 (0.9)	3 (1.2)	1.000
PE	0	1 (0.4)	0.430
CAD	0	1 (0.4)	0.430
Lung atelectasis	0	4 (1.6)	0.034 *
Pneumonia	0	10 (4.0)	<0.001 *
Hematoma	0	3 (1.2)	0.078
Surgical site infection	0	12 (4.8)	<0.001 *
Displacement of the implant	0	5 (2.0)	<0.001 *
PONV	123 (37.1)	80 (32.1)	0.253
LOS (days)	7 (6–9)	9 (8–12)	<0.001 †
Hospitalization cost ($)	1958 (933–2966)	2815 (1019–3555)	<0.001 †
Readmission in 30 days	5 (1.5)	5 (2.0)	0.751

Note: Data are presented as frequency (percentage), or median (IQR). * *p*-value from Fisher’s exact test; † *p*-value from Wilcoxon rank-sum test. *p* < 0.05 indicates statistical significance. Abbreviations: SD (standard deviation), PE (pulmonary embolism), CAD (coronary artery disease), PONV, (postoperative nausea and vomiting), LOS (length of stay).

## Data Availability

Data sets generated or analyzed in this study are accessible upon reasonable request from the corresponding author.

## Supplementary Materials

The following supporting information can be downloaded at https://www.mdpi.com/article/10.3390/jcm15135307/s1, Table S1: Comparison of Maximum Postoperative NRS Pain Scores Within the First 96 Hours Between EA and DA Groups; Table S2: Sensitivity analysis of postoperative outcomes among patients undergoing ≥ 3 level instrumented lumbar fusion (*n* = 219); Table S3: Univariable Analysis of Factors Associated with Early Ambulation Following patients undergoing ≥ 3 level instrumented lumbar fusion; Table S4: Multivariable Analysis of Factors Associated with Early Ambulation Following patients undergoing ≥ 3 level instrumented lumbar fusion.

## Abbreviations

EA	Early Ambulation
DA	Delayed Ambulation
ERAS	Enhanced Recovery After Surgery
ASA class	American Society of Anesthesiologists class
ORs	Odds Ratio
RRs	Risk Ratio
LOS	Length of Stay
MSSIC	Michigan Spine Surgery Improvement Collaborative
IRB	Institutional Review Board
ICD-10	International Classification of Diseases 10
MIS	Minimally Invasive Surgery
FBSS	Failed Back Surgery Syndrome
INR	International Normalized Ratio
ECG	electrocardiography
ETCO2	End-Tidal Carbon Dioxide
BMI	Body Mass Index
PACU	Post-Anesthesia Care Unit
SpO2	Oxygen Saturation
NRS	Numerical Rating Scale
MME	Milligrams of IV Morphine Equivalent
GCS	Glasglow Coma Score
COPD	Chronic Obstructive Pulmonary Disease
CAD	Coronary Artery Disease
CKD	Chronic Kidney Disease
PONV	Postoperative Nausea and Vomiting
PE	Pulmonary Embolism
SD	Standard Deviation
IQR	Interquartile Range
CI	Confidence Interval
STROBE	Strengthening the Reporting of Observational Studies in Epidemiology
BG	Bone Graft
mL	Milliliter
IV	Intravenous
h	Hours
uOR	Univariable Odds Ratio
mOR	Multivariable Odds Ratio
BI	Barthel Index
SSEP	Somatosensory Evoked Potentials
MEP	Motor Evoked Potentials
ACS-NSQIP	The American College of Surgeons-National Surgical Quality Improvement Program
RCTs	Randomized Controlled Trials
