# Supplementary material for: Factors Associated with the Early Ambulation After Elective Lumbar Spinal Surgery: A Retrospective Cohort Study
Source: J Clin Med. 2026 Jul 7;15(13):5307. doi: 10.3390/jcm15135307 (PMC13362772; doi:10.3390/jcm15135307)
Supplement: Supplementary file 1 [file jcm-15-05307-s001.zip › jcm-4338220-supplementary.pdf]

Supplement

Diagnostic and Procedural Classification

Diagnostic classifications were identified according to the *International Classification of Diseases, 10th Revision (ICD–10)*. The following diagnostic codes were included:

- M48.06 (spinal stenosis, lumbar region)
- M48.07 (spinal stenosis, lumbosacral region)
- M43.16 (spondylolisthesis, lumbar region)
- M51.1 (lumbar intervertebral disc disorder with radiculopathy)

Corresponding surgical procedures were identified using *ICD–9–-CM procedure codes*, including:

- 81.08 (lumbar and lumbosacral fusion of the anterior column, posterior technique)
- 03.09 (exploration and decompression of the spinal canal)
- 80.51 (excision of intervertebral disc; discectomy)
- 77.79 (excision of bone for graft at vertebrae)

Table S1. Comparison of Maximum Postoperative NRS Pain Scores Within the First 96 Hours Between EA and DA Groups

Time Point	EA group (*n* = 332))	DA group (*n* = 249)	p-value
Maximum NRS at 24 h	4 (2–7)	7 (6–8)	<0.001*
Maximum NRS at 48 h	3 (2–4)	7 (5–8)	<0.001*
Maximum NRS at 72 h	3 (2–4)	5 (4–6)	<0.001*
Maximum NRS at 96 h	3 (2–4)	5 (4–6)	<0.001*

Median (IQR): *p-values (Wilcoxon rank-sum test), <0.05 significant, **Abbreviations**: NRS (Numerical Rating Scale Rating Scale), h (Hours)

Table S2. Sensitivity analysis of postoperative outcomes among patients undergoing ≥ 3 level instrumented lumbar fusion **(*n* = 219)**

Associated factors and Outcomes data	EA group (*n* = 106)	DA Group (*n* = 113)	P-value
Age (years old)	60.22±7.20	62.60±7.33	0.016†
Male	22 (20.75)	38 (33.63)	0.035*
ASA class 1-2	86 (81.13)	70 (61.95)	0.002*
Operation time (minutes)	275 (195–355)	330 (250–410)	0.001
Anesthetic time (minutes)	300 (220–380)	350 (270–435)	<0.001‡
Blood loss (mL)	500 (450–800)	500 (500–1500)	<0.001‡
Received IV parecoxib administration	65 (61.32)	13 (11.50)	<0.001*
**Outcomes data**			
Amount of opioid used in 96 h (MME)	8 (7–10)	11 (9–15)	<0.001‡
P.O. drain in 96 h (mL)	192.64±82.77	202.21±93.96	0.426
Overall Complications	70 (66.04)	91 (80.53)	0.021*
LOS (days)	8 (6–11)	10 (8–12)	0.015*
Hospitalization cost ($)	3,629.3 (2,927.1–5,132.0)	4,195.6 (3,238.6–6,233.8)	<0.001‡
Readmission in 30 days	2 (1.89)	1 (0.88)	0.612

Note: Data are presented as frequency (percentage), mean ± SD, or median (IQR). * p-value from Fisher's exact test; † p-value from Student's t-test; ‡ p-value from Wilcoxon rank-sum test. $p < 0.05$ indicates statistical significance. **Abbreviations**: SD (Standard Deviation), ASA (American Society of Anesthesiologist), mL (milliliter), IV (Intravenous), MME (milligram of intravenous morphine equivalents), P.O. (Postoperative). LOS (length of stay).

Table S3. Univariable Analysis of Factors Associated with Early Ambulation Following patients undergoing ≥3 level instrumented lumbar fusion.

Associated factors	uOR	95% CI of Odds Ratio	P-value
Age ≤ 65 years	1.37	0.75–2.50	0.316
Male	0.52	0.27–0.99	0.035*
ASA class 1-2	2.64	1.37–5.18	0.002*
Anesthetic time < 300 minutes	3.27	1.82–5.92	<0.001*
Intraoperative EBL < 500 mL	1.28	0.73–2.26	0.417
Postoperative intravenous parecoxib use	12.20	5.82–26.50	<0.001*

Note: * $p < 0.05$ indicates statistical significance. **Abbreviations**: uOR (univariable odds ratio), CI (Confidence Interval), ASA (American Society of Anesthesiologists), BG (bone graft), EBL (estimated blood loss), mL (milliliter).

Table S4. Multivariable Analysis of Factors Associated with Early Ambulation Following patients undergoing ≥3 level instrumented lumbar fusion.

Associated factors	mOR	95% CI of Odds Ratio	P-value
Age ≤ 65 years	1.12	0.53–2.34	0.765
Male	0.54	0.25–1.17	0.117
ASA class 1-2	2.27	1.02–5.03	0.045*
Anesthetic time < 200 minutes	2.59	1.32–5.06	0.005*
Intraoperative EBL < 500 mL	1.00	0.51–1.94	0.993
Postoperative intravenous parecoxib use	12.36	5.88–26.06	<0.001*

Note: * $p < 0.05$ indicates statistical significance. **Abbreviations**: mOR (multivariable odds ratio), CI (Confidence Interval), ASA (American Society of Anesthesiologists), BG (bone graft), EBL (estimated blood loss), mL (milliliter).
